# Association of dietary patterns with sarcopenia in adults aged 50 years and older

**DOI:** 10.1007/s00394-024-03370-6

**Published:** 2024-04-03

**Authors:** Elisa Mazza, Yvelise Ferro, Samantha Maurotti, Francesca Micale, Giada Boragina, Raffaella Russo, Lidia Lascala, Angela Sciacqua, Carmine Gazzaruso, Tiziana Montalcini, Arturo Pujia

**Affiliations:** 1 Department of Clinical and Experimental Medicine, University Magna Grecia, 88100 Catanzaro, Italy; 2 Department of Medical and Surgical Science, University Magna Grecia, 88100 Catanzaro, Italy; 3 Research Center for the Prevention and Treatment of Metabolic Diseases, University Magna Grecia, 88100 Catanzaro, Italy; 4 Diabetes and Endocrine-Metabolic Diseases Unit, Istituto Clinico Beato Matteo, Gruppo Ospedaliero San Donato, Corso Pavia 84, 27029 Vigevano, Italy; 5 Department of Biomedical Sciences for Health, University of Milan, Milan, Italy

**Keywords:** Handgrip strength, Sarcopenia, Principal components analysis, Adults, Dietary patterns, Mediterranean diet

## Abstract

**Purpose:**

Although numerous studies have investigated the impact of dietary factors on the prevention of decreased muscle mass and function, limited research has examined the relationship between dietary patterns and sarcopenia. This study aimed to assess the associations between dietary patterns, and sarcopenia, muscle strength, and mass in adults following a Mediterranean diet residing in southern Italian cities.

**Methods:**

This cross-sectional study utilized data from an existing database, comprising 528 individuals aged 50 years or older who underwent health-screening tests at the Clinical Nutrition Unit of the “R.Dulbecco” University Hospital. Strength was assessed through handgrip strength, and appendicular skeletal muscle mass was estimated using bioelectrical impedance analysis. Dietary intake information was collected through a food frequency questionnaire linked to the MetaDieta 3.0.1 nutrient composition database. Principal Component Analysis, a statistical technique identifying underlying relationships among different nutrients, was employed to determine dietary patterns. Multinomial logistic regression analysis was conducted to estimate the odds ratio for sarcopenia or low handgrip strength in relation to the lowest tertile of dietary pattern adherence compared to the highest adherence.

**Results:**

The participants had a mean age of 61 ± 8 years. Four dietary patterns were identified, with only the Western and Mediterranean patterns showing correlations with handgrip strength and appendicular skeletal muscle mass. However, only the Mediterranean pattern exhibited a correlation with sarcopenia (r =  − 0.17, p = 0.02). The highest tertile of adherence to the Mediterranean dietary pattern demonstrated significantly higher handgrip strength compared to the lowest tertile (III Tertile: 28.3 ± 0.5 kg vs I Tertile: 26.3 ± 0.5 kg; p = 0.01). Furthermore, even after adjustment, the highest tertile of adherence to the Mediterranean pattern showed a significantly lower prevalence of sarcopenia than the lowest adherence tertile (4% vs 16%, p = 0.04). The lowest adherence to the Mediterranean dietary pattern was associated with increased odds of having low muscle strength (OR = 2.38; p = 0.03; 95%CI = 1.05–5.37) and sarcopenia (OR = 9.69; p = 0.0295; %CI = 1.41–66.29).

**Conclusion:**

A high adherence to the Mediterranean dietary pattern, characterized by increased consumption of legumes, cereals, fruits, vegetables, and limited amounts of meat, fish, and eggs, is positively associated with handgrip strength and appendicular skeletal muscle mass. The highest adherence to this dietary model is associated with the lowest odds of low muscle strength and sarcopenia. Despite the changes brought about by urbanization in southern Italy compared to the past, our findings continue to affirm the superior benefits of the Mediterranean diet in postponing the onset of frailty among older adults when compared to other dietary patterns that are rich in animal foods.

**Supplementary Information:**

The online version contains supplementary material available at 10.1007/s00394-024-03370-6.

## Introduction

Sarcopenia is a clinical condition characterized by a decline in skeletal muscle mass and function, which is associated with various risk factors such as physical inactivity, aging, malnutrition, smoking, diabetes, and social factors [1]. The consequences of sarcopenia include physical disabilities, reduced quality of life, depression, and even mortality [2]. Sarcopenia has also been linked to an increased risk of hospitalization [3], cardiovascular and respiratory diseases, cognitive impairment [4], osteoporosis, and fractures [5].

Currently, there are no approved specific drugs for treating sarcopenia. Non-pharmacological approaches such as resistance exercise and proper nutrition are the current management options [4]. Many studies investigating the role of diet in sarcopenia have focused on individual nutrients that affect muscle mass. For example, it has been demonstrated that a high intake of salt can lead to fat accumulation and muscle weakness associated with sarcopenia [6].

Moreover, an increased intake of saturated fatty acids has been linked to heightened susceptibility to muscle weakness [7], mediated through pathways involving inflammatory pathways, including NF-κB activation, leading to elevated levels of pro-inflammatory cytokines such as TNF-α and IL-6, as well as oxidative stress, and insulin resistance [8]. These mechanisms have the potential to impede mitochondrial function and hinder muscle protein synthesis [8]. In contrast, a diet rich in mono and polyunsaturated fatty acids (MUFAs and PUFAs) among the elderly has shown to improve physical performance compared to a low-fat diet [9].

In the Health ABC Study cohort, participants in the highest quintile of animal protein intake experienced approximately 40% less lean mass loss over the 3-year follow-up period compared to those in the lowest quintile [10]. This could be explained because the consumption of animal proteins provides a broad spectrum of essential amino acids, including branched-chain amino acids such as leucine, which are particularly effective in stimulating muscle protein synthesis [11].

However, sarcopenia is influenced by multiple dietary factors, including both excessive and inadequate nutrient intake, which collectively affect the risk of the disease over time. Emerging evidence suggests that overall dietary patterns, rather than individual nutrients or foods, may provide better insights into clinical outcomes [12]. Nutrients are consumed as part of a mixture, and their physiological effects can be influenced by interactions with other nutrients, other foods consumed during a meal, or the foods that deliver them [12]. Rather than focusing on isolated nutrients or foods, dietary pattern analysis examines the impact of the overall diet.

While there is sufficient evidence confirming a relationship between adherence to healthy dietary patterns and intermediate markers of sarcopenia, there is currently a lack of definitive evidence regarding its association with sarcopenia itself [13]. The role of the Mediterranean diet in preventing sarcopenia remains uncertain. However, a recent systematic review [14] demonstrated the positive effects of the Mediterranean dietary pattern on muscle mass and function.

To definitively establish whether the Mediterranean diet is associated with sarcopenia, and not just its surrogates, it is important to investigate this association in the population residing in the area where the Mediterranean diet originated. The “traditional” Mediterranean diet is a plant-based dietary pattern that was prevalent in the Mediterranean region, as observed in the late 1950s and early 1960s in rural villages of Southern Italy, such as Nicotera, and Greece, as part of the renowned Seven Countries Study. Ancel Keys demonstrated that the population in small towns of southern Italy exhibited better health outcomes compared to affluent individuals in urban areas of the United States, largely due to their specific dietary pattern. Although the dietary profile of southern Italy has retained its fundamental characteristics, and vital statistics continue to confirm the advantages of this eating pattern in Mediterranean countries [15], diets in Mediterranean countries are gradually becoming modernized and influenced by Western dietary habits. Additionally, race and ethnicity are associated with differential intakes of food groups and nutrients [16, 17], and the rate of muscle decline varies with age and across populations [18, 19].

Therefore, our aim was to investigate, through a large cross-sectional study conducted on a population of adults residing in southern Italian cities near the village of Nicotera, whether there is an association between the current dietary pattern and sarcopenia.

## Population and methods

This cross-sectional study was conducted from February 2019 to January 2023 and received protocol approval from the local ethics committee at the “Mater Domini” University Hospital in Catanzaro, Italy (now “R. Dulbecco”). The hospital is located approximately 55.9 miles (90 km) away from Nicotera. The project was approved by the Local Ethic Committee (no. 21.04.2022–123/CE). All participants signed written informed consent. The investigation conforms to the ethical principles outlined in the Declaration of Helsinki. The study population comprised 528 subjects consecutively referred to the clinical nutrition outpatient clinic (aged 50–91 years) residing in Calabria, southern Italy. These individuals were undergoing health-screening tests at the Clinical Nutrition Unit of the “Mater Domini” University Hospital in Catanzaro, Italy.

We used the data available from a pre-existing database. Exclusion criteria were applied to individuals with severe chronic clinical conditions of the kidney, and liver and who were unable to undergo strength measurements due to rheumatic diseases, malignant tumors, physical disabilities and NYHA functional class ≥ II patients. Patients who were taking dietary supplements or medications that could influence muscle strength or the risk of falls, such as psychotropic drugs, glucocorticoids, sex hormones, growth hormone, thyroid hormone, and protein supplements (e.g., whey protein, branched-chain amino acids, essential amino acids), as indicated in their clinical records, were also excluded.

### Dietary intake assessment

Dietary intake data were collected using a food frequency questionnaire (FFQ) to gather information on the frequency and portion sizes of food and beverage consumption over the past month. In this study, the FFQ was administered by an interviewer (dietitian) rather than being self-administered. To account for known systematic errors in FFQs and to ensure internal calibration, a less biased short-term instrument, that is a 24-h recall (24HR), was also administered. The FFQ involved combining portion size information with frequency data by asking participants to estimate their usual consumption amount in terms of specified units [20]. Portion sizes were based on typical or natural servings (e.g., a slice of bread, one egg), and when a standard portion size was not obvious, a commonly used size was selected (e.g., one cup). To enhance reporting accuracy, our questionnaires included portion size images. All the collected data were linked to a nutrient composition database called MetaDieta 3.0.1 (San Benedetto del Tronto, Italy). This database provided information on nutrient intake [20]. The dietary intake calculations were primarily based on the INRAN (National Institute of Food Research) 2000 and IEO (European Institute of Oncology) 2008 and 2015 databases, as well as the CREA (Centro di Ricerca alimenti e nutrizione) 2019 database. Additionally, the USDA (Department of Agriculture) database was used for the oxygen radical absorbance capacity (ORAC) values of selected foods and the USDA Nutrient database. The Metadieta database contains information on over 6,500 food items and up to 150 food components, and it is updated annually. This software also translates foods and beverages into major food group equivalents such as fish, meat, cereals, fruits, legumes, eggs, milk, potatoes, vegetables, sugary drinks, animal fats/margarines, and cake/pies. The adherence to Mediterranean diet was assessed using the Mediterranean diet score. Total score ranges from 0 (minimum adherence) to 55 (maximum adherence). A score from 25 to 55 indicates a moderate-high adherence to this eating pattern [21].

### Anthropometric assessments

Body weight (BW) data were obtained from medical records, and body mass index (BMI) was calculated as weight (kg) divided by height (m) squared. Obesity was defined as a BMI ≥ 30 kg/m^2^.

Hand-to-foot bioelectrical impedance analysis (BIA) data were available for a subgroup of 170 individuals (aged 50–91) to estimate appendicular skeletal muscle mass (ASMM) using the manufacturer's equations (Akern, Bodygram Plus software) [22]. ASMM represents the combined muscle mass of the arms and legs. In accordance with the criteria set by the European Working Group on Sarcopenia in Older People (EWGSOP2) [1], a cutoff value of 15 kg for women and 20 kg for men was used to diagnose low ASMM based on BIA measurements.

### Muscular strength

Handgrip strength (HGS) of the dominant hand was assessed using a handgrip dynamometer (manufactured by SAEHAN Corporation, Masan-Korea) [23]. Three maximal isometric contractions were performed for each strength test, with each contraction lasting 3 s. The average of the three trials was used as the criterion score.

### Sarcopenia assessment

Sarcopenia was diagnosed according to the criteria established by the European Working Group on Sarcopenia in Older People (EWGSOP2) [1]. The diagnosis required the presence of both low muscle strength, measured as HGS below 16 kg for women and below 27 kg for men, and low muscle mass, assessed as ASMM by BIA with cutoff values of less than 15 kg for women and less than 20 kg for men.

### Physical activity assessment

Participants’ physical activity levels were assessed using the validate NPAQ-short questionnaire [24]. The participants were categorized into two groups: those engaged in moderate/vigorous physical activity (MVPA) and those with sedentary behavior or engaged in light physical activity. MVPA activities included activities such as brisk walking, dancing, gardening, sports/exercise, and walking domestic animals [25]. Following the approach suggested by Lee et al. [26], we considered older adults to be engaged in MVPA even when walking at their usual pace. This data allowed us to determine whether inactive participants were more likely to develop sarcopenia and experience a decline in muscle strength.

### Biochemical evaluation

Data from medical records were utilized for the biochemical evaluation. Venous blood samples were collected after an overnight fast and processed within 4 h. Serum levels of glucose, creatinine, total cholesterol, alanine transaminase (ALT), aspartate transaminase (AST), γ glutamyl transferase (γGT), were measured using the Roche Cobas Electrochemiluminescent Immunoassay (COBAS 6000, Roche, Switzerland). Quality control assessments were performed daily for all measurements.

### Statistical analysis

#### Food patterns analysis

Principal Component Analysis (PCA) is a statistical technique utilized to reduce the dimensionality of a dataset while preserving essential information [20, 27]. PCA helps identify underlying structures and relationships among different nutrients. The objective of using PCA was to simplify data analysis and identify the primary contributors to dietary variations. The process of implementing PCA for food pattern analysis involves several steps. *Assessing the relationship between variables*: The initial step involves examining the relationships between variables in the dataset, which, in this case, are individual nutrients. Typically, correlation coefficients are calculated to determine significant relationships, with variables having correlation coefficients above 0.4 considered relevant. *Constructing the covariance matrix:* Using the selected correlated variables, a covariance matrix is constructed. This matrix summarizes the correlations between all possible pairs of variables, providing a measure of the strength and direction of these relationships. *Transforming variables into principal components*: The original variables (nutrients) are transformed into new variables known as principal components (PCs). Each PC is a linear combination of the initial variables. This transformation ensures that the PCs are uncorrelated with each other. The objective is to identify a smaller set of PCs that capture most of the variation present in the original dataset. *Orthogonal rotation:* After obtaining the PCs, an orthogonal rotation technique is applied to enhance their interpretability. The varimax rotation is employed, as it maximizes the variance of the loadings (weights) of the original variables on each PC. This rotation simplifies the interpretation of resulting food patterns. *Deriving food patterns*: Each PC represents a direction within the multidimensional nutrient space that exhibits the most variance. The first PC accounts for the largest possible variance in the dataset. By examining the loadings (weights) of the original variables on each PC, one can identify the nutrients that contribute most to that particular component. These nutrients can be interpreted as representing specific food patterns or dietary factors. A higher absolute value in the correlation coefficients indicates that a nutrient has a stronger contribution to the construction of the principal component (PC). To determine the number of PCs to retain, a scree plot of the eigenvalues derived from the correlation matrix of the standardized variables is examined. The eigenvalue represents the proportion of variance explained by each component. According to the Kaiser criterion, the number of components to retain in PCA is equal to the number of eigenvalues greater than 1. Eigenvalues and eigenvectors are always paired, meaning that each eigenvector has a corresponding eigenvalue. The number of eigenvectors/eigenvalues is equal to the number of dimensions in the dataset. By ranking the eigenvectors in descending order based on their eigenvalues, we can determine the significance of the PCs. The eigenvector with the highest eigenvalue represents the most important PC, while eigenvectors with insignificant eigenvalues are ignored. In the final step, the data is reoriented from the original axes to the ones represented by the PCs obtained through PCA. This transformation allows for a more meaningful interpretation of the data in terms of the identified PCs.

#### Association between sarcopenia/muscle mass parameters and food patterns

After conducting a varimax rotation, PC scores representing the weighted sums of the exposure variables were generated to represent the dietary patterns. Pearson's correlation test was employed to identify correlations between the dietary patterns and muscle mass parameters such as ASMM, HGS and sarcopenia, considering that the continuous variables followed a normal distribution. The dietary patterns were then categorized into tertiles of adherence (high, medium, and low) to assess the association between food patterns and sarcopenia, ASMM, HGS, and low HGS, thereby providing sensitive indicators of nutrient intake levels.

General characteristics of study population across tertiles of adherence to dietary patterns were examined using analysis of variance (ANOVA) for continuous variables or chi-square test for categorical variables. Adjusted sarcopenic indices (ASMM and HGS) and sarcopenia alone across tertiles of adherence to dietary patterns were calculated using analysis of covariance (ANCOVA). To determine the association between adherence to dietary patterns and sarcopenia and its components, multinomial logistic regression analysis was used in crude model and full-adjusted model in which all the possible confounders (including age, gender, BMI, physical activity, calories, protein intake and use of lipid lowering medications) were adjusted. The overall trend of odds ratios (ORs) across tertiles was calculated by considering the tertiles of adherence to dietary patterns as ordinal variables.

Finally, sensitivity analysis was conducted for individuals aged 65 years or older (we performed again we performed PCA, Pearson’s correlation, ANOVA, ANCOVA and χ2 tests). All statistical tests were two-sided, with a significance level set at p < 0.05. The statistical analyses were performed using SPSS 25.0 for Windows (IBM Corporation, New York, NY, USA).

## Results

The study population had a mean age of 61 ± 8 years, with 62% of participants being female. Among the participants, 11% had low HGS, and sarcopenia was observed in 9% of individuals (assessed in a subgroup of 170 participants). The characteristics of the study population are presented in Table 1. The energy, nutrient, and food group intakes are summarized in Table 2. The mean energy intake was 2028 ± 542 kcal. Additionally, the mean protein intake per kilogram of body weight per day was 1.0 ± 0.3 g.

**Table 1:** Mean ± SD participants’anthropometric, and clinical characteristics

Variables	Mean	SD
Age (years)	61	8
BMI (Kg/m^2^)	30	4
HGS (Kg)	28	10
ASMM (Kg)	18.2	4.2
Glucose (mg/dL)	98	22
Creatinine (mg/dL)	0.83	0.2
TC (mg/dL)	195	42
AST (IU/L)	24	14
ALT (IU/L)	26	22
γGT (UI/L)	31	30
Prevalence		
Gender (women, %)	62	
Smokers (%)	25	
MV physical activity (%)	45	
Obesity (%)	44	
Hypertension (%)	56	
Antihypertensive agents (%)	53	
Hyperlipidemia (%)	52	
Lipid-lowering agents (%)	32	
T2DM (%)	13	
Oral antidiabetic agents (%)	10	
Low HGS (%)	11	
Low ASMM (%)^*^	28	
Sarcopenia (%)^*^	9	

*BMI* body mass index, *HGS* handgrip strength, *ASMM* appendicular skeletal muscle mass, *TC* total cholesterol, *AST* aspartate aminotransferase, *ALT* alanine aminotransferase, *γGT* γ glutamyltransferase, *MV* Moderate/Vigorous, *T2DM* type 2 diabetes mellitus

^*^Low ASMM and sarcopenia diagnosis in only 170 participants

**Table 2:** Mean ± SD participants’ energy, nutrients and food group intake

Variables	Mean	SD
Energy intake (kcal/day)	2028	542
Animal protein (g/day)	46	18
Plant protein (g/day)	29	10
Animal lipids (g/day)	31	15
Plant lipids (g/day)	52	21
Saturated fatty acids (g/day)	25	9
Monounsaturated fatty acids (g/day)	45	16
Polyunsaturated fatty acids (g/day)	12	5
Cholesterol (mg/day)	237	105
Carbohydrates (g/day)	244	78
Soluble fiber (g/day)	4	2
Insoluble fiber (g/day)	10	4
Food groups		
Milk and dairy products (servings/day)	1.7	1.0
Meat, fish and eggs (servings/day)	1.5	0.8
Legumes (servings/day)	0.2	0.2
Cereals (servings/day)	3.1	1.3
Vegetables (servings/day)	1.8	1.0
Fruit (servings/day)	2.2	1.4
Cakes/pies (servings/day)	1.9	1.4
Adherence to the MD (score)	30	3

*MD* Mediterranean Diet

**Fig. 1:**
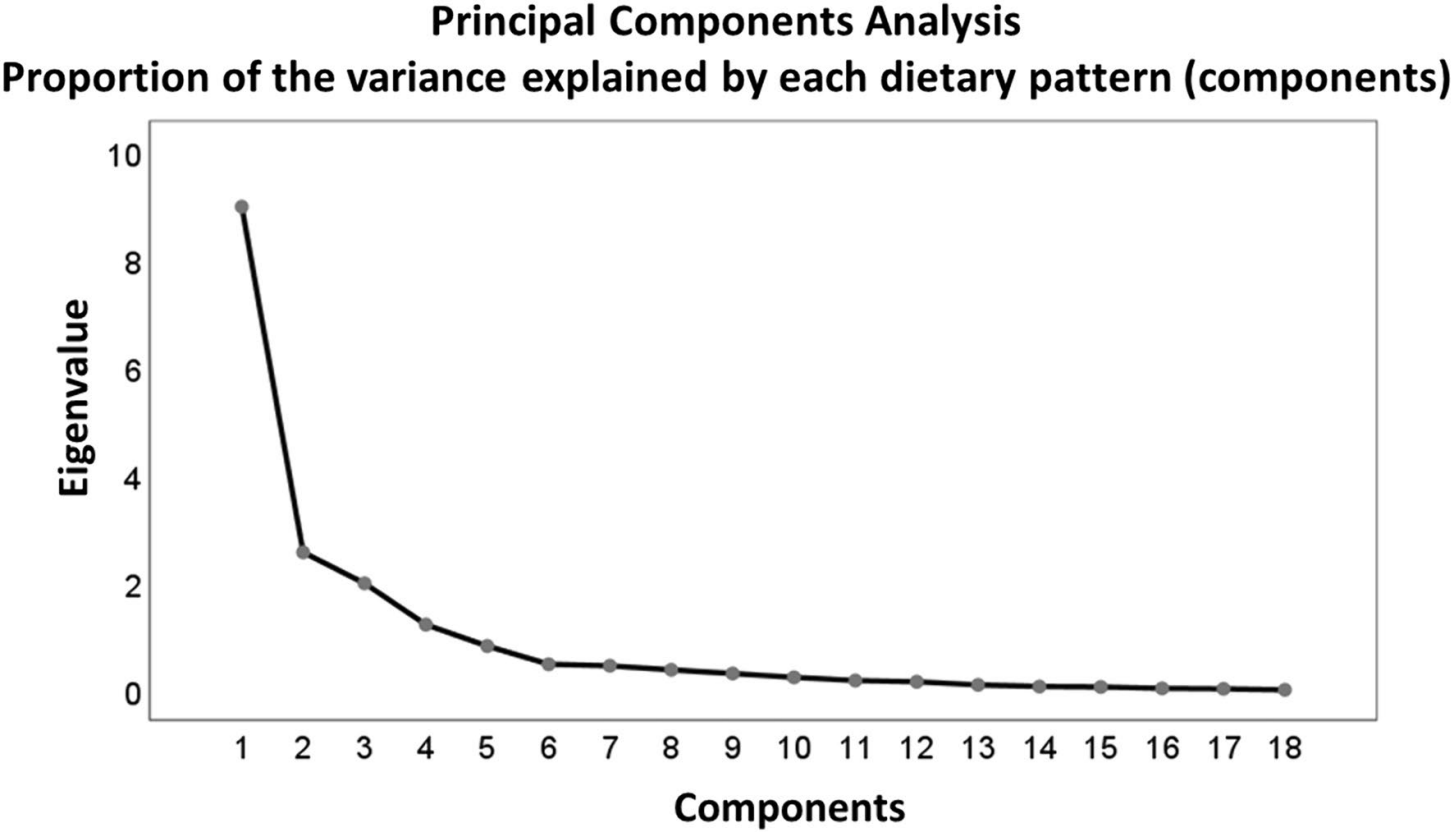
Food patterns derived from the PCA

Figure 1 presents the food patterns obtained through PCA. Four distinct dietary patterns were identified, collectively explaining 82% of the variance in nutrient composition. These patterns are as follows: 1. Western (This pattern is characterized by high consumption of carbohydrates, animal proteins, animal fats, saturated fatty acids, and cholesterol); 2. Mediterranean (The Mediterranean pattern is characterized by a higher intake of carbohydrates, plant proteins, and fiber); 3. High Fats: (greater consumption of plant fats, saturated fats, and mono/polyunsaturated fats); 4. Carnivorous (high consumption of animal proteins and cholesterol). Table S1 in the Supplementary Material shows the factor loadings derived from principal component analysis conducted with dietary variables.

The correlations between dietary patterns and HGS, ASMM and sarcopenia are shown in Table 3. In particular Western pattern was positive correlated with HGS and ASMM; Mediterranean pattern exhibited a stronger positive correlation with HGS and a higher positive correlation with ASMM, and a negative correlation with sarcopenia (Table 3). No significant associations were found between any other dietary patterns (High Fats and Carnivorous) and the muscle parameters or sarcopenia (Table 3).

**Table 3:** Univariate analyses—dietary pattern correlated with the handgrip strength, Appendicular skeletal muscle mass and Sarcopenia

Western pattern	HGS	ASMM	Sarcopenia
*r*	0.16	0.14	− 0.04
*p*	p < 0.001	0.06	0.53
Mediterranean pattern	HGS	ASMM	Sarcopenia
*r*	0.26	0.31	− 0.17
*p*	< 0.001	< 0.001	0.02
High Fat	HGS	ASMM	Sarcopenia
*r*	0.030	0.12	− 0.06
*p*	0.49	0.11	0.38
Carnivorous	HGS	ASMM	Sarcopenia
*r*	− 0.06	0.31	0.02
*p*	0.16	0.85	0.73

*HGS* handgrip strength, *ASMM* appendicular skeletal muscle mass

Table 4 presents the multivariable-adjusted mean HGS across tertiles of the two dietary patterns. A significant positive linear trend was observed for HGS across increasing tertiles of both the Western and Mediterranean patterns (p-trend = 0.001 and < 0.001, respectively). In the highest tertile of the Mediterranean pattern, after adjusting for non-dietary factors, physical activity, and lipid-lowering medications (in the case of the Western pattern), there was a significantly higher HGS compared to the lowest tertile (Tertile 3: HGS 28.3 ± 0.5 kg vs Tertile 1: 26.3 ± 0.5 kg; p = 0.01). However, no significant association was found between the Western pattern and HGS (p = 0.4; Table 4). A positive linear trend was also observed for ASMM across increasing tertiles of both the Western and Mediterranean dietary patterns (p-trend = 0.05 and 0.001, respectively).

**Table 4:** Multivariable-adjusted means of handgrip strength and appendicular skeletal muscle mass across tertiles of dietary patterns—General Linear Model test

Dietary pattern	I Tertile (low level) (n = 176)	II Tertile (medium level) (n = 176)	III Tertile (high level) (n = 176)	*p-value*	*Post-Hoc Analysis p-value*
Western dietary pattern					
HGS (kg)	26.3 ± 10	26.8 ± 9	29.8 ± 10	0.001	1 vs 2 0.004 2 vs 3 0.017
Model 1	28.2 ± 0.6	27.2 ± 0.5	27.5 ± 0.6	0.41	/
Mediterrean dietary pattern					
HGS (kg)	24.5 ± 9	28.3 ± 11	30.2 ± 10	< 0.001	1 vs 2 0.001 1 vs 3 < 0.001
Model 1	26.3 ± 0.5	28.2 ± 0.5	28.3 ± 0.5	0.010	1 vs 2 0.007 1 vs 3 0.010

Dietary pattern	I Tertile (low level) (n = 56)	II Tertile (medium level) (n = 57)	III Tertile (high level) (n = 57)	*p-value*	*Post-Hoc analysis p-value*
Western dietary pattern					
ASMM (kg)	17.5 ± 3	17.8 ± 4	19.3 ± 5	0.05	2 vs 3 0.07
Model 1^*^	18.3 ± 0.3	18.3 ± 0.3	17.9 ± 0.4	0.69	/
Mediterrean dietary pattern					
ASMM (kg)	16.5 ± 3	19.0 ± 4	19.1 ± 5	0.001	1 vs 2 0.005 1 vs 3 0.004
Model 1^*^	17.7 ± 0.3	18.3 ± 0.3	18.6 ± 0.3	0.16	1 vs 3 0.06

*HGS* handgrip strength, *ASMM* appendicular skeletal muscle mass

Model 1: Adjusted for age, gender, BMI, physical activity, dietary energy, daily protein intake per kg of body weight, physical activity (only for Mediterranean pattern) and lipid-lowering agents (only for Western pattern)

^*^Model 1: Adjusted for age, gender, obesity, dietary energy and daily protein intake per kg of body weight

Tables S3 and S5in the Supplementary Material provide detailed information on the main characteristics of the tertiles within each dietary pattern.

In particular, in the Table S3 the highest tertile of the Mediterranean dietary pattern (III Tertile) was characterized by a higher tendency to consume of legumes (0.3 ± 0.2 portions/day; p < 0.001), cereals (3.7 ± 1.4 portions/day; p < 0.001), vegetables (2.4 ± 1.1 portions/day; p < 0.001) and fruit (3.3 ± 1.5 portions/day; p < 0.001) and a lower quantity of meat, fish and eggs (1.7 ± 0.9 portions/day; p = 0.001) compared to lowest tertile, underlining the distinctive characteristics of the Mediterranean dietary model compared to the Western pattern rich in milk and dairy products, cakes and poor in vegetables (Table S5).

**Fig. 2:**
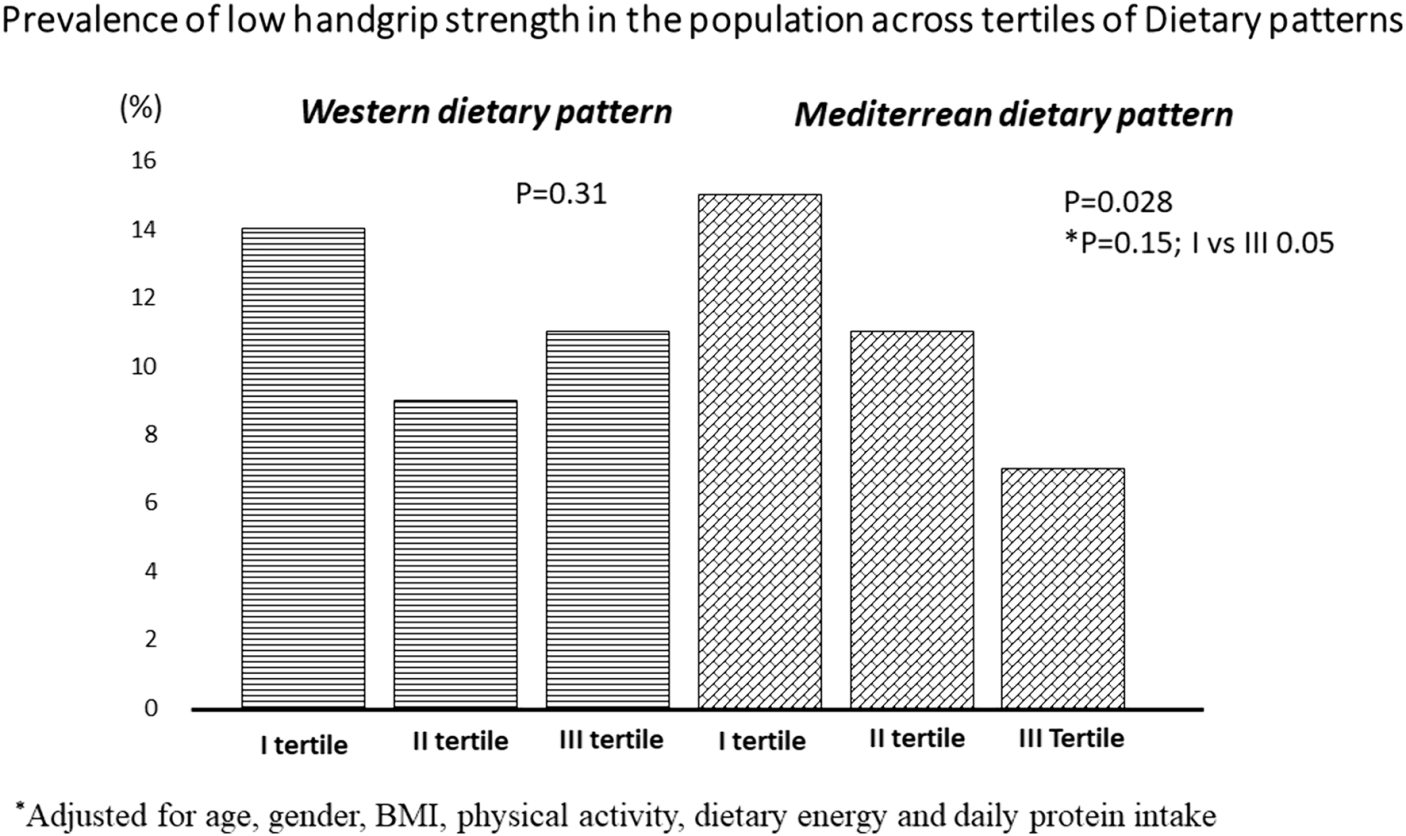
Prevalence of low handgrip strength in the population across tertiles of Dietary patterns

**Fig. 3:**
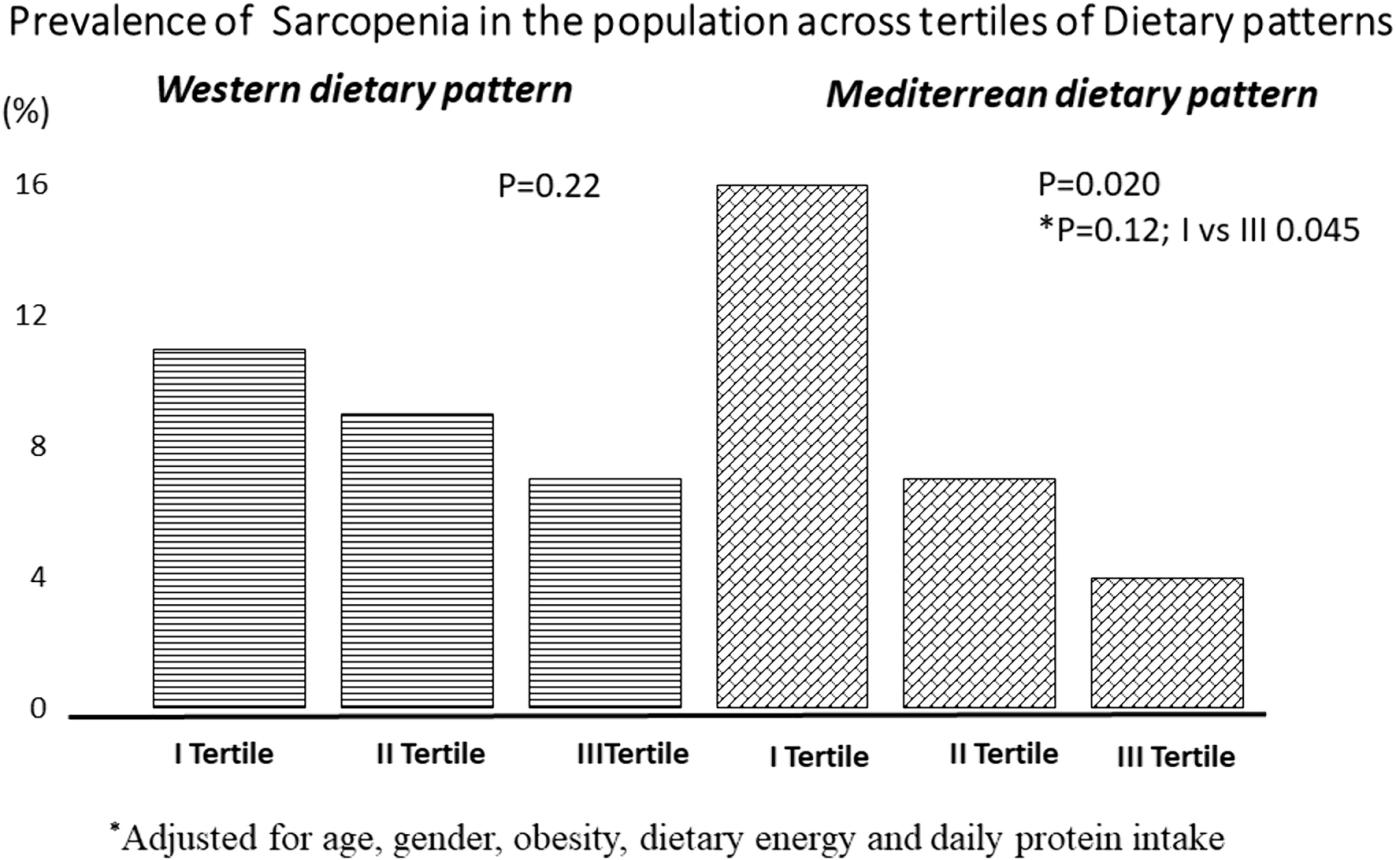
Prevalence of sarcopenia in the population across tertiles of Dietary patterns

Figure 2 and Fig. 3 display the prevalence of low HGS and sarcopenia, respectively, according to the tertiles of the Mediterranean dietary pattern. It is noteworthy that even after adjusting for confounding factors, the highest tertile of the Mediterranean pattern had a significantly lower prevalence of low HGS (7% vs. 15%, p = 0.05) and sarcopenia (4% vs. 16%, p = 0.04).

The multinomial logistic regression analysis revealed that the Mediterranean dietary pattern maintained an association with sarcopenia and HGS (Table 5). Specifically, a lower adherence to the Mediterranean dietary pattern was significantly associated with increased odds of having low muscle strength (B = 0.86; p = 0.03; odds ratio [OR] = 2.38; confidence interval [CI] 1.05–5.37) and sarcopenia (B = 2.27; p = 0.02; OR = 9.69, CI 1.41–66.29) when compared to the highest adherence to this dietary pattern, which represented the lowest risk.

**Table 5:** Multinomial logistic regression analysis “Sarcopenia” and “Low handgrip strength” being the dependent binary variables, adjusted predictions with 95% CI

Dependent binary variable “*Sarcopenia*”	B	SE	*p-value*	OR	95% CI	
					LL	UL
Mediterrean dietary pattern						
I Tertile	2.27	0.98	0.021	9.69	1.41	66.29
II Tertile	1.30	1.01	0.19	3.69	0.50	26.91
III Tertile	–	–	–	–	–	–
Age	0.05	0.02	0.06	1.05	0.99	1.11
Gender	0.64	0.71	0.36	1.91	0.47	7.71
Obesity	− 2.47	1.07	0.021	0.08	0.01	0.68
Daily protein intake per kg of BW	1.73	1.02	0.09	5.69	0.76	42.47

Dependent binary variable “*Low HGS*”	B	SE	*p-value*	OR	95% CI	
					LL	UL
Mediterrean dietary pattern						
I Tertile	0.86	0.41	0.036	2.38	1.05	5.37
II Tertile	0.54	0.39	0.16	1.73	0.79	3.77
III Tertile	–	–	–	–	–	–
Age	0.07	0.01	< 0.001	1.07	1.04	1.11
Gender	– 0.10	0.32	0.74	0.90	0.48	1.68
BMI	0.02	0.03	0.52	1.02	0.95	1.09
Physical activity	− 0.06	0.29	0.81	0.93	0.52	1.66
Daily protein intake per kg of BW	0.77	0.49	0.12	2.16	0.81	5.72

*HG* handgrip strength, *ASMM* appendicular skeletal muscle mass, *B* unstandardized coefficient, *SE* standard error, *OR* odds ratio, *CI* confidence interval, *LL* lower limit, *UL* upper limit, *BW* body weight, *HGS* handgrip strength, *BMI* body mass index

**Fig. 4:**
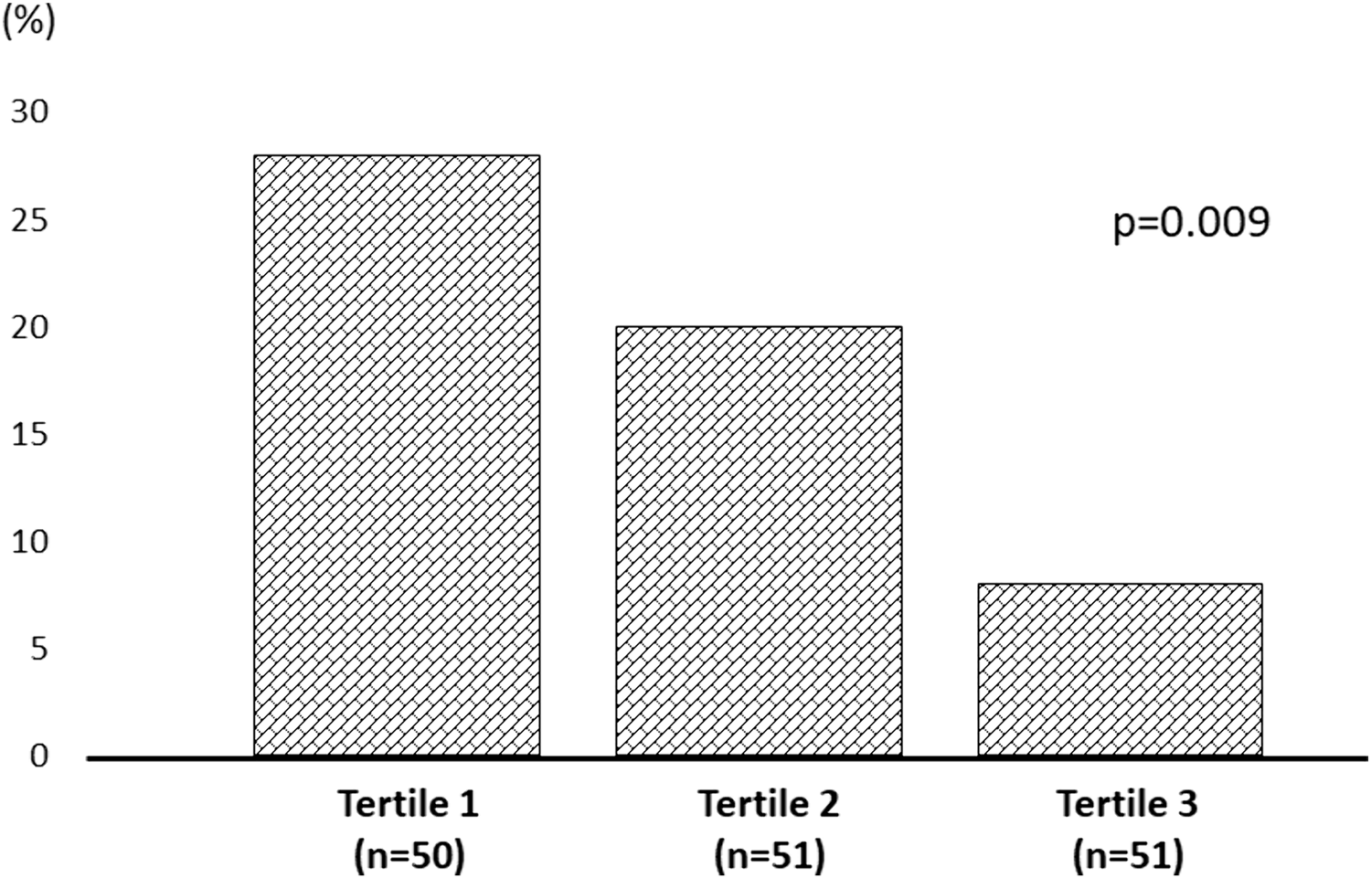
Prevalence of low handgrip strength in the elderly population across tertiles of Mediterrean dietary pattern

In the subgroup analysis of 152 elderly subjects aged 65 years or older, we observed a significant correlation between the Mediterranean dietary pattern and HGS (r = 0.44; p < 0.001) as well as ASMM (r = 0.31; p = 0.007). The anthropometric and clinical characteristic, energy and nutrients and food groups intake across tertile of Mediterranean pattern in elderly population were shown in Supplemental Table 6 and 7. Figure 4 showed that the highest tertile of adherence to the Mediterranean diet exhibited a significantly lower prevalence of “low HGS” compared to the lowest tertile (8% vs. 20%, p = 0.009).

## Discussion

In this large cohort of adults from a Mediterranean area, our findings support previous research demonstrating that a higher adherence to a Mediterranean dietary pattern is associated with a lower prevalence of sarcopenia, including its individual component (low HGS). We also observed a significant and positive linear trend for ASMM and HGS across increasing tertiles of the Mediterranean dietary pattern, with a stronger association seen in the elderly population. No significant associations were found between the Western dietary pattern or other dietary patterns and HGS, ASMM, or sarcopenia.

Even in non-Mediterranean countries, increased compliance with a Mediterranean dietary pattern has been linked to protection against chronic diseases [28]. While there have been some investigations into the effects of different diets on muscle mass, HGS, and appendicular lean mass, studies in this area are still limited, particularly when it comes to sarcopenia.

Some studies have suggested a significant association between various healthy dietary patterns and gait speed, an intermediate marker of sarcopenia risk [13]. However, in a cross-sectional study involving middle-aged adults, dietary patterns that included both healthy and unhealthy foods did not influence muscle strength [29]. A systematic review and meta-analysis of observational studies indicated that higher protein intake was associated with greater physical functioning in older adults [30] while another meta-analysis focusing on non-frail and non-sarcopenic adults demonstrated only marginal effects of additional protein intake combined with resistance exercise on lean body mass and lower body strength [31]. It is worth noting that a recent systematic review [14] highlighted the positive effects of the Mediterranean dietary pattern on muscle mass and function. This review included both cross-sectional and prospective studies, with a predominant focus on older participants. In a prospective population-based study conducted in Tuscany, Italy, which was part of the InCHIANTI Study, higher adherence to the Mediterranean diet was associated with better lower body performance [32]. However, in two studies specifically examining sarcopenia, no association between diet and sarcopenia was observed [33, 34]. Analyzing data from the Nurses' Health Study involving 71,941 women aged 60 years and older, adherence to the Mediterranean diet, Dietary Approaches to Stop Hypertension (DASH) diet, or Alternate Healthy Eating Index-2010 (AHEI-2010) was associated with a 13%, 7%, and 10% lower risk of frailty, respectively [35]. Lower consumption of red and processed meat, and higher intake of monounsaturated fats and plant-based food varieties were independently associated with a reduced risk of frailty [36, 37].

Our results are consistent with previous studies, but we expand upon them by establishing an association between the Mediterranean diet and sarcopenia, rather than relying solely on indirect measures of muscle mass and function. In contrast to the Health ABC Study [10], we did not find a relationship between a Carnivorous dietary pattern (high in animal protein) and high HGS or muscle mass. This result is not surprising, as the effects of macronutrients cannot be considered in isolation, as they vary depending on the replacement nutrient and the specific foods providing them [12]. Focusing solely on individual nutrients in a reductionist manner often fails to consider the substitution effects with other nutrients and associated foods. This can explain the differences between the two studies.

The dietary pattern of southern Italy in the 1960s, as discovered in the Nicotera village by Keys, has been considered a genuine representation of the Mediterranean Italian diet [38]. This traditional dietary model of southern Italy and its associated health benefits have retained their basic features, as supported by vital statistics in Mediterranean countries [14].

While the composition of the Mediterranean diet remains relatively consistent, there may be some country-specific variations influenced by local cultures, traditions, and urbanization [39]. Nevertheless, the Mediterranean diet predominantly remains a plant-based diet with limited consumption of animal products. In comparison to a typical Western diet, total protein intake in the traditional Mediterranean diet is approximately 20% lower, with the Western diet being higher in animal protein. Legumes, nuts, seeds, and whole grains are the primary sources of protein in the Mediterranean diet, while animal protein sources include fish, chicken, eggs, and small amounts of lean meats and dairy products [40]. Current evidence suggests that the overall Mediterranean dietary pattern, rather than specific individual foods or components, provides the most substantial benefits. However, certain components of the Mediterranean diet, such as plant proteins, polyunsaturated fatty acids (PUFAs), monounsaturated fatty acids (MUFAs), antioxidants, and other micronutrients, play important roles in the association between the diet and sarcopenia. It is likely that protein quality may be more important that quantity in mediating the beneficial effects of the Mediterranean diet on muscle mass [41]. Plant proteins, in particular, have been linked to a lower risk of frailty [42] while PUFAs, MUFAs, and antioxidants have been associated with a reduced inflammatory state and protection against age-related muscle damage [43, 44]. Additionally, n-3 PUFAs have demonstrated anabolic effects by activating the mTORC1 signaling pathway in muscle [45], which is essential for muscle growth. Amino acids, such as leucine, and energy deprivation can also influence mTORC1 activity [46]. The InCHIANTI study revealed a positive association between dietary intake of antioxidants and physical performance measures [47]. Compared to Western-type dietary patterns, the Mediterranean diet exhibits greater diversity in food plant varieties, races, species, and subspecies. Consistent with these findings, our study indicates a protective effect of plant foods compared to less healthy animal foods on the risk of muscle mass loss and sarcopenia.

Our study suggests that a higher adherence to a Mediterranean dietary pattern, which is primarily plant-based, is associated with a lower prevalence of sarcopenia in older adults compared to a low adherence to this eating model. The study highlights the importance of the quality of the diet and supports a global shift towards healthful plant-based diets to mitigate sarcopenia, particularly in older adults.

Among the strengths of this study is its approach to assessing dietary patterns rather than focusing on single nutrients, which allows it to capture the complex relationship between diet and sarcopenia, providing a more comprehensive understanding of the topic.

However, there are some limitations to consider. The study only assessed dietary intake at a single time point, which may not fully capture long-term dietary exposures. As an observational study, it cannot establish a causal relationship between dietary intake and changes in muscle mass or the development of sarcopenia. Despite adjusting for multiple confounders, there may still be residual confounding from unmeasured factors, including other dietary components. The study’s inclusion criteria may have excluded individuals with a higher probability of having sarcopenia, leading to an underestimation of its prevalence in the study population.

Finally, while the study supports the benefits of a Mediterranean dietary approach, primarily focused on plant-based foods, in preventing sarcopenia and frailty in older adults, further research is needed to better understand the causal mechanisms and to address the limitations of this study. Nonetheless, the findings suggest that promoting healthful plant-based diets can have significant health benefits.

## Conclusion

In this study, we found that individuals who followed a Mediterranean dietary pattern, which mainly consists of plant-based foods, had a lower prevalence of sarcopenia compared to those who had a low adherence to this eating pattern. These results emphasize the significance of the quality of one's diet and support the global trend towards adopting healthful plant-based diets, particularly among older adults, as a means of preventing frailty.

Given that the prevention of sarcopenia has become a major objective for public health experts and healthcare providers, adopting a Mediterranean dietary approach could have substantial health and economic advantages.

### Supplementary Information

Below is the link to the electronic supplementary material.

Supplementary file1 (DOCX 43 KB)

## Data Availability

The datasets in the present study can be obtained from the corresponding author upon a reasonable request.

## References

[1] Cruz-Jentoft AJ, Bahat G, Bauer J, Boirie Y, Bruyere O, Cederholm T, Cooper C, Landi F, Rolland Y, Sayer AA et al (2019) Sarcopenia: revised european consensus on definition and diagnosis. Age Ageing 48:16–31 30312372 10.1093/ageing/afy169 PMC6322506

[2] Beaudart C, Rizzoli R, Bruyere O, Reginster J, Biver E (2014) Sarcopenia: burden and challenges for public health. Arch. Public. Health 72:45–45 (**eCollection 2014**) 25810912 10.1186/2049-3258-72-45 PMC4373245

[3] Cruz-Jentoft AJ, Landi F, Topinkova E, Michel J (2010) Understanding sarcopenia as a geriatric syndrome. Curr Opin Clin Nutr Metab Care 13:1–7 19915458 10.1097/MCO.0b013e328333c1c1

[4] Montalcini T, Pujia A, Donini LM, Frittitta L, Galvano F, Natali A, Pironi L, Porrini M, Riso P, Rivellese AA et al (2020) A call to action: now is the time to screen elderly and treat osteosarcopenia, a position paper of the italian college of academic nutritionists MED/49 (ICAN-49). Nutrients 12:2662. 10.3390/nu12092662 32878316 10.3390/nu12092662 PMC7550989

[5] Yeung SSY, Reijnierse EM, Pham VK, Trappenburg MC, Lim WK, Meskers CGM, Maier AB (2019) Sarcopenia and its association with falls and fractures in older adults: a systematic review and meta-analysis. J Cachexia Sarcopenia Muscle 10:485–500 30993881 10.1002/jcsm.12411 PMC6596401

[6] Yoshida Y, Kosaki K, Sugasawa T, Matsui M, Yoshioka M, Aoki K, Kuji T, Mizuno R, Kuro-O M, Yamagata K et al (2020) High salt diet impacts the risk of sarcopenia associated with reduction of skeletal muscle performance in the Japanese population. Nutrients 12:3474. 10.3390/nu12113474 33198295 10.3390/nu12113474 PMC7696631

[7] Arias-Fernandez L, Struijk EA, Rodriguez-Artalejo F, Lopez-Garcia E, Lana A (2020) Habitual dietary fat intake and risk of muscle weakness and lower-extremity functional impairment in older adults: a prospective cohort study. Clin Nutr 39:3663–3670 32273201 10.1016/j.clnu.2020.03.018

[8] Martins AR, Nachbar RT, Gorjao R, Vinolo MA, Festuccia WT, Lambertucci RH, Cury-Boaventura MF, Silveira LR, Curi R, Hirabara SM (2012) Mechanisms underlying skeletal muscle insulin resistance induced by fatty acids: importance of the mitochondrial function. Lipids Health Dis 11:30 22360800 10.1186/1476-511X-11-30 PMC3312873

[9] Poggiogalle E, Rossignon F, Carayon A, Capel F, Rigaudiere J, De Saint Vincent S, Le-Bacquer O, Salles J, Giraudet C, Patrac V et al (2022) Deleterious effect of high-fat diet on skeletal muscle performance is prevented by high-protein intake in adult rats but not in old rats. Front Physiol 12:749049 35111075 10.3389/fphys.2021.749049 PMC8801536

[10] Houston DK, Nicklas BJ, Ding J, Harris TB, Tylavsky FA, Newman AB, Lee JS, Sahyoun NR, Visser M, Kritchevsky SB et al (2008) Dietary protein intake is associated with lean mass change in older, community-dwelling adults: the health, aging, and body composition (Health ABC) study. Am J Clin Nutr 87:150–155 18175749 10.1093/ajcn/87.1.150

[11] Volpi E, Kobayashi H, Sheffield-Moore M, Mittendorfer B, Wolfe RR (2003) Essential amino acids are primarily responsible for the amino acid stimulation of muscle protein anabolism in healthy elderly adults. Am J Clin Nutr 78:250–258 12885705 10.1093/ajcn/78.2.250 PMC3192452

[12] Tapsell LC, Neale EP, Satija A, Hu FB (2016) Foods, nutrients, and dietary patterns: interconnections and implications for dietary guidelines. Adv Nutr 7:445–454 27184272 10.3945/an.115.011718 PMC4863273

[13] Van Elswyk ME, Teo L, Lau CS, Shanahan CJ (2022) Dietary patterns and the risk of sarcopenia: a systematic review and meta-analysis. Curr Dev Nutr 6:nzac001 35542386 10.1093/cdn/nzac001 PMC9071101

[14] Papadopoulou SK, Detopoulou P, Voulgaridou G, Tsoumana D, Spanoudaki M, Sadikou F, Papadopoulou VG, Zidrou C, Chatziprodromidou IP, Giaginis C et al (2023) Mediterranean diet and sarcopenia features in apparently healthy adults over 65 years: a systematic review. Nutrients 15:1104. 10.3390/nu15051104 36904104 10.3390/nu15051104 PMC10005300

[15] Ferro-Luzzi A, Branca F (1995) Mediterranean diet, Italian-style: prototype of a healthy diet. Am J Clin Nutr 61:1338S-1345S 7754985 10.1093/ajcn/61.6.1338S

[16] Dietary Guidelines Advisory Committee. 2020. Scientific Report of the 2020 Dietary Guidelines Advisory Committee: Advisory Report to the Secretary of Agriculture and the Secretary of Health and Human Services. U.S. Department of Agriculture, Agricultural Research Service, Washington, DC. https://www.Dietaryguidelines.Gov. Accessed 30 June 2023

[17] Syddall H, Evandrou M, Cooper C, Sayer AA (2009) Social inequalities in grip strength, physical function, and falls among community dwelling older men and women: findings from the hertfordshire cohort study. J Aging Health 21:913–939 19597159 10.1177/0898264309340793

[18] Mitchell WK, Williams J, Atherton P, Larvin M, Lund J, Narici M (2012) Sarcopenia, dynapenia, and the impact of advancing age on human skeletal muscle size and strength; a quantitative review. Front Physiol 3:260 22934016 10.3389/fphys.2012.00260 PMC3429036

[19] Gallagher D, Visser M, De Meersman RE, Sepulveda D, Baumgartner RN, Pierson RN, Harris T, Heymsfield SB (1985) Appendicular skeletal muscle mass: effects of age, gender, and ethnicity. J Appl Physiol 83:229–239 10.1152/jappl.1997.83.1.229 9216968

[20] Colica C, Mazza E, Ferro Y, Fava A, De Bonis D, Greco M, Foti DP, Gulletta E, Romeo S, Pujia A et al (2017) Dietary patterns and fractures risk in the elderly. Front Endocrinol (Lausanne) 8:344 29375472 10.3389/fendo.2017.00344 PMC5770658

[21] Panagiotakos DB, Pitsavos C, Stefanadis C (2006) Dietary patterns: a Mediterranean diet score and its relation to clinical and biological markers of cardiovascular disease risk. Nutr Metab Cardiovasc Dis 16:559–568 17126772 10.1016/j.numecd.2005.08.006

[22] Buscemi C, Ferro Y, Pujia R, Mazza E, Boragina G, Sciacqua A, Piro S, Pujia A, Sesti G, Buscemi S et al (2021) Sarcopenia and appendicular muscle mass as predictors of impaired fasting glucose/type 2 diabetes in elderly women. Nutrients 13:1909. 10.3390/nu13061909 34199375 10.3390/nu13061909 PMC8227668

[23] Montalcini T, Migliaccio V, Yvelise F, Rotundo S, Mazza E, Liberato A, Pujia A (2013) Reference values for handgrip strength in young people of both sexes. Endocrine 43:342–345 22752930 10.1007/s12020-012-9733-9

[24] Danquah IH, Petersen CB, Skov SS, Tolstrup JS (2018) Validation of the NPAQ-Short—a brief questionnaire to monitor physical activity and compliance with the WHO recommendations. BMC Public Health 18:601-y 29739383 10.1186/s12889-018-5538-y PMC5941676

[25] World Health Organization. WHO- what is Moderate-Intensity and Vigorous-Intensity Physical Activity? WHO.2017. http://www.Who.Int/Dietphysicalactivity/Physical_activity_intensity/En/. Accessed 30 June 2023

[26] Lee I, Shiroma EJ, Kamada M, Bassett DR, Matthews CE, Buring JE (2019) Association of step volume and intensity with all-cause mortality in older women. JAMA Intern Med 179:1105–1112 31141585 10.1001/jamainternmed.2019.0899 PMC6547157

[27] Mazza E, Fava A, Ferro Y, Moraca M, Rotundo S, Colica C, Provenzano F, Terracciano R, Greco M, Foti D et al (2017) Impact of legumes and plant proteins consumption on cognitive performances in the elderly. J Transl Med 15:109–115 28532453 10.1186/s12967-017-1209-5 PMC5440936

[28] Woo J, Woo KS, Leung SS, Chook P, Liu B, Ip R, Ho SC, Chan SW, Feng JZ, Celermajer DS (2001) The mediterranean score of dietary habits in chinese populations in four different geographical areas. Eur J Clin Nutr 55:215–220 11305271 10.1038/sj.ejcn.1601150

[29] Samadi M, Khosravy T, Azadbakht L, Rezaei M, Mosafaghadir M, Kamari N, Bagheri A, Pasdar Y, Najafi F, Hamze B et al (2021) Major dietary patterns in relation to muscle strength status among middle-aged people: a cross-sectional study within the RaNCD cohort. Food Sci Nutr 9:6672–6682 34925797 10.1002/fsn3.2617 PMC8645754

[30] Coelho-Junior HJ, Milano-Teixeira L, Rodrigues B, Bacurau R, Marzetti E, Uchida M (2018) Relative protein intake and physical function in older adults: a systematic review and meta-analysis of observational studies. Nutrients 10:1330. 10.3390/nu10091330 30235845 10.3390/nu10091330 PMC6163569

[31] Nunes EA, Colenso-Semple L, McKellar SR, Yau T, Ali MU, Fitzpatrick-Lewis D, Sherifali D, Gaudichon C, Tome D, Atherton PJ et al (2022) Systematic review and meta-analysis of protein intake to support muscle mass and function in healthy adults. J Cachexia Sarcopenia Muscle 13:795–810 35187864 10.1002/jcsm.12922 PMC8978023

[32] Milaneschi Y, Bandinelli S, Corsi AM, Lauretani F, Paolisso G, Dominguez LJ, Semba RD, Tanaka T, Abbatecola AM, Talegawkar SA et al (2011) Mediterranean diet and mobility decline in older persons. Exp Gerontol 46:303–308 21111801 10.1016/j.exger.2010.11.030 PMC3056167

[33] Isanejad M, Sirola J, Mursu J, Rikkonen T, Kroger H, Tuppurainen M, Erkkila AT (2018) Association of the baltic sea and mediterranean diets with indices of sarcopenia in elderly women, OSPTRE-FPS study. Eur J Nutr 57:1435–1448 28303397 10.1007/s00394-017-1422-2

[34] Chan R, Leung J, Woo JA (2016) Prospective cohort study to examine the association between dietary patterns and sarcopenia in Chinese community-dwelling older people in Hong Kong. J Am Med Dir Assoc 17:336–342 26774365 10.1016/j.jamda.2015.12.004

[35] Struijk EA, Hagan KA, Fung TT, Hu FB, Rodriguez-Artalejo F, Diet L-G (2020) Quality and risk of frailty among older women in the nurses’ health study. Am J Clin Nutr 111:877–883 32091575 10.1093/ajcn/nqaa028 PMC7138663

[36] Struijk EA, Fung TT, Sotos-Prieto M, Rodriguez-Artalejo F, Willett WC, Hu FB, Lopez-Garcia E (2022) Red meat consumption and risk of frailty in older women. J Cachexia Sarcopenia Muscle 13:210–219 34755477 10.1002/jcsm.12852 PMC8818608

[37] Fung TT, Struijk EA, Rodriguez-Artalejo F, Willett WC, Lopez-Garcia E (2020) Fruit and vegetable intake and risk of frailty in women 60 years old or older. Am J Clin Nutr 112:1540–1546 33022693 10.1093/ajcn/nqaa256 PMC7727483

[38] Helsing E (1995) Traditional diets and disease patterns of the Mediterranean, Circa 1960. Am J Clin Nutr 61:1329S-1337S 7754984 10.1093/ajcn/61.6.1329S

[39] Dernini S, Berry EM, Serra-Majem L, La Vecchia C, Capone R, Medina FX, Aranceta-Bartrina J, Belahsen R, Burlingame B, Calabrese G et al (2017) Med diet 4.0: the Mediterranean diet with four sustainable benefits. Public Health Nutr 20:1322–1330 28003037 10.1017/S1368980016003177 PMC10261651

[40] Herforth A, Arimond M, Álvarez-Sánchez C, Coates J, Christianson K, Muehlhoff E (2019) A global review of food-based dietary guidelines. Adv Nutr 10:590–605 31041447 10.1093/advances/nmy130 PMC6628851

[41] Tosti V, Bertozzi B, Fontana L (2018) Health benefits of the mediterranean diet: metabolic and molecular mechanisms. J Gerontol A Biol Sci Med Sci 73:318–326 29244059 10.1093/gerona/glx227 PMC7190876

[42] Struijk EA, Fung TT, Rodriguez-Artalejo F, Bischoff-Ferrari HA, Hu FB, Willett WC, Lopez-Garcia E (2022) Protein intake and risk of frailty among older women in the nurses’ health study. J Cachexia Sarcopenia Muscle 13:1752–1761 35318829 10.1002/jcsm.12972 PMC9178161

[43] Dalle S, Rossmeislova L, Koppo K (2017) The role of inflammation in age-related sarcopenia. Front Physiol 8:1045 29311975 10.3389/fphys.2017.01045 PMC5733049

[44] Sakuma K, Aoi W, Yamaguchi A (2015) Current understanding of sarcopenia: possible candidates modulating muscle mass. Pflugers Arch 467:213–229 24797147 10.1007/s00424-014-1527-x

[45] Yoshino J, Smith GI, Kelly SC, Julliand S, Reeds DN, Mittendorfer B (2016) Effect of dietary N-3 PUFA supplementation on the muscle transcriptome in older adults. Physiol Rep 4:e12785. 10.14814/phy2.12785 27252251 10.14814/phy2.12785 PMC4908485

[46] Deldicque L, Theisen D, Francaux M (2005) Regulation of mTOR by amino acids and resistance exercise in skeletal muscle. Eur J Appl Physiol 94:1–10 15702344 10.1007/s00421-004-1255-6

[47] Cesari M, Pahor M, Bartali B, Cherubini A, Penninx BW, Williams GR, Atkinson H, Martin A, Guralnik JM, Ferrucci L (2004) Antioxidants and physical performance in elderly persons: the Invecchiare in Chianti (InCHIANTI) study. Am J Clin Nutr 79:289–294 14749236 10.1093/ajcn/79.2.289

