# Supplementary material for: Association of dietary patterns with sarcopenia in adults aged 50 years and older
Source: Eur J Nutr. 2024 Apr 3;63(5):1651–62. doi: 10.1007/s00394-024-03370-6 (PMC11329607; doi:10.1007/s00394-024-03370-6)
Supplement: Supplementary file 1 — Supplementary file1 (DOCX 43 KB) [file 394_2024_3370_MOESM1_ESM.docx]

| **Supplemental Table 1.** *Mean ± SD participants’anthropometric and clinical characteristics across tertiles of Mediterranean pattern* | | | | | |
| --- | --- | --- | --- | --- | --- |
| Variables | Tertile 1  (low level) (n=176) | Tertile 2  (medium level) (n=176) | Tertile 3  (high level)  (n=176) | *p-value* | *P-post hoc analysis* |
| Age (years) | 62±9 | 61±8 | 61±7 | 0.44 | / |
| BMI (Kg/m^2^) | 29.1±5 | 29.7±4 | 30.1±4 | 0.08 | 1 vs 3 0.027 |
| Glucose (mg/dL) | 99±25 | 97±19 | 99±21 | 0.55 | / |
| Creatinine (mg/dL) | 0.80±0.2 | 0.84±0.2 | 0.84±0.2 | 0.11 | / |
| TC (mg/dL) | 197±42 | 194±40 | 193±44 | 0.69 | / |
| AST (IU/L) | 24±12 | 22±11 | 25±17 | 0.30 | / |
| ALT (IU/L) | 25±18 | 24±13 | 27±19 | 0.24 | / |
| γGT (UI/L) | 33±34 | 27±20 | 30±30 | 0.35 | / |
| *Prevalence* | | | | | |
| Gender (women, n; %) | (132)75 | (109) 62 | (86) 49 | <0.001 | / |
| Smokers (%) | 24 | 24 | 27 | 0.44 | / |
| MI Physical activity (%) | 39 | 40 | 56 | 0.001 | / |
| Obesity (%) | 41 | 44 | 48 | 0.16 |  |
| Hypertension (%) | 55 | 53 | 59 | 0.45 | / |
| Antihypertensive agents (%) | 53 | 48 | 57 | 0.52 | / |
| Hyperlipidemia (%) | 49 | 51 | 56 | 0.24 | / |
| Lipid-lowering agents (%) | 27 | 33 | 36 | 0.10 | / |
| T2DM (%) | 14 | 14 | 14 | 0.89 | / |
| Oral antidiabetic agents (%) | 11 | 13 | 12 | 0.90 | / |
| BMI, body mass index; HG, hand grip; ASMM, appendicular skeletal muscle mass; TC, total cholesterol; AST, aspartate aminotransferase; ALT, alanine aminotransferase, γGT, gamma glutamyltransferase; MI, Moderate-intensity; T2DM, type 2 diabetes mellitus. | | | | | |

| **Supplemental Table 2.** *Mean ± SD participants’ energy, nutrients and food groups intake across tertiles of Mediterranean pattern* | | | | | |  |
| --- | --- | --- | --- | --- | --- | --- |
| Variables | Tertile 1  (low level) (n=176) | Tertile 2  (medium level) (n=176) | Tertile 3  (high level)  (n=176) | *p-value* | *P-post hoc analysis* |  |
| Energy intake (kcal/day) | 1747±423 | 2007±492 | 2332±538 | <0.001 | 1 vs 2 <0.001  1 vs 3 <0.001  2 vs 3 <0.001 |  |
| Animal protein (g/day) | 42±17 | 46±18 | 48±20 | 0.008 | 1 vs 2 0.05  1 vs 3 0.002 |  |
| Plant protein (g/day) | 21±5 | 29±6 | 38±8 | <0.001 | 1 vs 2 <0.001  1 vs 3 <0.001  2 vs 3 <0.001 |  |
| Animal lipids (g/day) | 30±15 | 31±15 | 31±15 | 0.84 | / |  |
| Plant lipids (g/day) | 50±20 | 49±19 | 58±23 | <0.001 | 1 vs 3 0.001  2 vs 3 <0.001 |  |
| Saturated fatty acids (g/day) | 24±9 | 25±9 | 25±9 | 0.78 | / |  |
| Monounsaturated fatty acids (g/day) | 44±15 | 42±15 | 47±16 | 0.026 | 2 vs 3 0.007 |  |
| Polyunsaturated fatty acids (g/day) | 10±4 | 11±4 | 13±6 | <0.001 | 1 vs 2 0.048  1 vs 3 <0.001  2 vs 3 <0.001 |  |
| Cholesterol (mg/day) | 228±99 | 229±94 | 253±113 | 0.037 | 1 vs 3 0.023  2 vs 3 0.030 |  |
| Carbohydrates (g/day) | 189±52 | 246±68 | 297±73 | <0.001 | 1 vs 2 <0.001  1 vs 3 <0.001  2 vs 3 <0.001 |  |
| Soluble fiber (g/day) | 2±1 | 3±1 | 5±2 | <0.001 | 1 vs 2 <0.001  1 vs 3 <0.001  2 vs 3 <0.001 |  |
| Insoluble fiber (g/day) | 6±2 | 9±3 | 14±4 | <0.001 | 1 vs 2 <0.001  1 vs 3 <0.001  2 vs 3 <0.001 |  |
| Iron (mg/day) | 8±2 | 11±2 | 14±3 | <0.001 | 1 vs 2 <0.001  1 vs 3 <0.001  2 vs 3 <0.001 |  |
| Calcium (mg/day) | 768±339 | 905±374 | 968±342 | <0.001 | 1 vs 2 <0.001  1 vs 3 <0.001 |  |
| Sodium (mg/day) | 1775±783 | 2068±824 | 2240±824 | <0.001 | 1 vs 2 0.001  1 vs 3 <0.001  2 vs 3 0.046 |  |
| Potassium (mg/day) | 2443±581 | 3057±611 | 4088±902 | <0.001 | 1 vs 2 <0.001  1 vs 3 <0.001  2 vs 3 <0.001 |  |
| Zinc (mg/day) | 8±2 | 9±3 | 11±3 | <0.001 | 1 vs 2 <0.001  1 vs 3 <0.001  2 vs 3 <0.001 |  |
| Magnesium (mg/day) | 193±45 | 246±48 | 323±65 | <0.001 | 1 vs 2 <0.001  1 vs 3 <0.001  2 vs 3 <0.001 |  |
| Selenium (mg/day) | 33±19 | 35±15 | 40±20 | <0.001 | 1 vs 3 <0.001  2 vs 3 0.007 |  |
| *Food groups* | | | | | |  |
| Milk and dairy products (servings/day) | 1.6±1.0 | 1.8±0.9 | 1.8±1.1 | 0.05 | 1 vs 3 0.019 | |
| Meat, fish and eggs (servings/day) | 1.4±0.7 | 1.4±0.6 | 1.7±0.9 | 0.001 | 1 vs 3 0.002  2 vs 3 0.001 | |
| Legumes (servings/day) | 0.1±0.1 | 0.2±0.2 | 0.3±0.2 | <0.001 | 1 vs 2 <0.001  1 vs 3 <0.001  2 vs 3 <0.001 | |
| Cereals (servings/day) | 2.3±0.9 | 3.1±1.3 | 3.7±1.4 | <0.001 | 1 vs 2 <0.001  1 vs 3 <0.001  2 vs 3 <0.001 |  |
| Vegetables (servings/day) | 1.3±0.7 | 1.6±0.8 | 2.4±1.1 | <0.001 | 1 vs 2 0.001  1 vs 3 <0.001  2 vs 3 <0.001 |  |
| Fruit (servings/day) | 1.2±0.7 | 1.9±0.9 | 3.3±1.5 | <0.001 | 1 vs 2 <0.001  1 vs 3 <0.001  2 vs 3 <0.001 |  |
| Oils/Animal fats/margarines (servings/day) | 2.1±1.0 | 1.9±0.9 | 2.1±1.0 | 0.13 | / |  |
| Cakes/pies (servings/day) | 1.9±1.4 | 2.0±1.4 | 1.8±1.3 | 0.56 | / |  |
| Adherence to the MD (score) | 29±4 | 30±2 | 32±3 | <0.001 | 1 vs 2 <0.001  1 vs 3 <0.001  2 vs 3 0.003 |  |
| MD, Mediterranean Diet. | | | | | |  |

| **Supplemental Table 3.** *Mean ± SD participants’anthropometric and clinical characteristics across tertiles of Western pattern* | | | | | |
| --- | --- | --- | --- | --- | --- |
| Variables | Tertile 1  (low level) | Tertile 2  (medium level) | Tertile 3  (high level) | *p-value* | *P-post hoc analysis* |
| Age (years) | 63±9 | 61±8 | 59±7 | <0.001 | 1 vs 2 0.010  1 vs 3 <0.001  2 vs 3 0.018 |
| BMI (Kg/m^2^) | 28.9±5 | 29.9±4 | 30.0±4 | 0.021 | 1 vs 2 0.025  1 vs 3 0.011 |
| Glucose (mg/dL) | 99±22 | 100±27 | 96±17 | 0.40 | / |
| Creatinine (mg/dL) | 0.83±0.2 | 0.83±0.2 | 0.83±0.2 | 0.97 | / |
| TC (mg/dL) | 191±44 | 196±43 | 196±39 | 0.50 | / |
| AST (IU/L) | 23±14 | 25±15 | 24±11 | 0.41 | / |
| ALT (IU/L) | 22±14 | 26±18 | 27±18 | 0.034 | 1 vs 3 0.010 |
| γGT (UI/L) | 28±23 | 33±38 | 30±23 | 0.49 | / |
| *Prevalence* | | | | | |
| Gender (women, n; %) | (124) 70 | (114) 65 | (89) 51 | <0.001 | / |
| Smokers (%) | 23 | 23 | 30 | 0.14 | / |
| MI Physical activity (%) | 48 | 50 | 46 | 0.72 | / |
| Obesity (%) | 36 | 48 | 48 | 0.032 | / |
| Hypertension (%) | 57 | 54 | 56 | 0.74 | / |
| Antihypertensive agents (%) | 55 | 50 | 53 | 0.74 | / |
| Hyperlipidemia (%) | 54 | 57 | 47 | 0.16 | / |
| Lipid-lowering agents (%) | 38 | 34 | 24 | 0.006 | / |
| T2DM (%) | 18 | 11 | 12 | 0.09 | / |
| Oral antidiabetic agents (%) | 14 | 11 | 10 | 0.24 | / |
| BMI, body mass index; HG, hand grip; ASMM, appendicular skeletal muscle mass; TC, total cholesterol; AST, aspartate aminotransferase; ALT, alanine aminotransferase, γGT, gamma glutamyltransferase; MI, Moderate-intensity; T2DM, type 2 diabetes mellitus. | | | | | |

| **Supplemental Table 4.** *Mean ± SD participants’ energy, nutrients and food groups intake across tertiles of Western pattern* | | | | | |
| --- | --- | --- | --- | --- | --- |
| Variables | Tertile 1  (low level) (n=176) | Tertile 2  (medium level) (n=176) | Tertile 3  (high level)  (n=176) | *p-value* | *P-post hoc analysis* |
| Energy intake (kcal/day) | 1671±350 | 1983±421 | 2433±538 | <0.001 | 1 vs 2 <0.001  1 vs 3 <0.001  2 vs 3 <0.001 |
| Animal protein (g/day) | 34±12 | 43±13 | 61±17 | <0.001 | 1 vs 2 <0.001  1 vs 3 <0.001  2 vs 3 <0.001 |
| Plant protein (g/day) | 27±9 | 29±10 | 32±10 | <0.001 | 1 vs 2 0.014  1 vs 3 <0.001  2 vs 3 0.002 |
| Animal lipids (g/day) | 18±6 | 28±8 | 46±13 | <0.001 | 1 vs 2 <0.001  1 vs 3 <0.001  2 vs 3 <0.001 |
| Plant lipids (g/day) | 53±21 | 51±21 | 53±21 | 0.69 | / |
| Saturated fatty acids (g/day) | 17±4 | 23±6 | 33±8 | <0.001 | 1 vs 2 <0.001  1 vs 3 <0.001  2 vs 3 <0.001 |
| Monounsaturated fatty acids (g/day) | 41±14 | 43±15 | 50±16 | <0.001 | 1 vs 3 <0.001  2 vs 3 <0.001 |
| Polyunsaturated fatty acids (g/day) | 11±5 | 11±4 | 13±5 | <0.001 | 1 vs 3 <0.001  2 vs 3 0.001 |
| Cholesterol (mg/day) | 175±69 | 228±78 | 306±112 | <0.001 | 1 vs 2 <0.001  1 vs 3 <0.001  2 vs 3 <0.001 |
| Carbohydrates (g/day) | 199±55 | 242±65 | 291±83 | <0.001 | 1 vs 2 <0.001  1 vs 3 <0.001  2 vs 3 <0.001 |
| Soluble fiber (g/day) | 4±2 | 4±2 | 4±2 | 0.84 | / |
| Insoluble fiber (g/day) | 10±5 | 9±4 | 9±4 | 0.06 | 1 vs 2 0.049  1 vs 3 0.036 |
| Iron (mg/day) | 10±3 | 11±3 | 13±3 | <0.001 | 1 vs 2 <0.001  1 vs 3 <0.001  2 vs 3 <0.001 |
| Calcium (mg/day) | 658±228 | 817±227 | 1166±392 | <0.001 | 1 vs 2 <0.001  1 vs 3 <0.001  2 vs 3 <0.001 |
| Sodium (mg/day) | 1437±394 | 1930±482 | 2716±925 | <0.001 | 1 vs 2 <0.001  1 vs 3 <0.001  2 vs 3 <0.001 |
| Potassium (mg/day) | 2971±953 | 3162±989 | 3455±954 | <0.001 | 1 vs 3 <0.001  2 vs 3 0.005 |
| Zinc (mg/day) | 7±2 | 9±2 | 12±3 | <0.001 | 1 vs 2 <0.001  1 vs 3 <0.001  2 vs 3 <0.001 |
| Magnesium (mg/day) | 233±76 | 249±73 | 280±71 | <0.001 | 1 vs 2 0.046  1 vs 3 <0.001  2 vs 3 <0.001 |
| Selenium (mg/day) | 34±20 | 33±19 | 41±16 | <0.001 | 1 vs 3 <0.001  2 vs 3 <0.001 |
| *Food groups* | | | | | |
| Milk and dairy products (servings/day) | 1.3±0.8 | 1.6±0.9 | 2.3±1.1 | <0.001 | 1 vs 2 0.001  1 vs 3 <0.001  2 vs 3 <0.001 |
| Meat, fish and eggs (servings/day) | 1.2±0.5 | 1.4±0.7 | 1.9±0.9 | <0.001 | 1 vs 2 0.027  1 vs 3 <0.001  2 vs 3 <0.001 |
| Legumes (servings/day) | 0.2±0.2 | 0.2±0.2 | 0.2±0.2 | 0.73 | / |
| Cereals (servings/day) | 2.6±1.1 | 3.0±1.3 | 3.6±1.4 | <0.001 | 1 vs 2 0.007  1 vs 3 <0.001  2 vs 3 <0.001 |
| Vegetables (servings/day) | 2.0±1.1 | 1.7±1.0 | 1.6±0.9 | 0.004 | 1 vs 2 0.015  1 vs 3 0.001 |
| Fruit (servings/day) | 2.3±1.5 | 2.1±1.4 | 2.1±1.4 | 0.63 | / |
| Oils/Animal fats/margarines (servings/day) | 2.0±0.9 | 2.1±1.0 | 2.1±1.0 | 0.89 | / |
| Cakes/pies (servings/day) | 1.3±0.9 | 1.9±1.2 | 2.6±1.5 | <0.001 | 1 vs 2 <0.001  1 vs 3 <0.001  2 vs 3 <0.001 |
| Adherence to the MD (score) | 31±4 | 30±3 | 30±3 | 0.001 | 1 vs 2 <0.001  1 vs 3 0.002 |
| MD, Mediterranean Diet. | | | | | |

| **Supplemental Table 6.** *Mean ± SD participants’anthropometric and clinical characteristics across tertiles of Mediterranean pattern in the elderly population* | | | | | |
| --- | --- | --- | --- | --- | --- |
| Variables | Tertile 1  (low level)  (n=50) | Tertile 2  (medium level) (n=51) | Tertile 3  (high level)  (n=51) | *p-value* | *P-post hoc analysis* |
| Age (years) | 73±7 | 72±7 | 71±5 | 0.08 | / |
| BMI (Kg/m^2^) | 28.1±5 | 30.2±5 | 30.6±5 | 0.017 | 1 vs 3 0.024 |
| Glucose (mg/dL) | 102±38 | 99±13 | 103±17 | 0.71 | / |
| Creatinine (mg/dL) | 0.84±0.2 | 0.85±0.2 | 0.88±0.2 | 0.65 | / |
| TC (mg/dL) | 190±45 | 194±39 | 188±43 | 0.75 | / |
| AST (IU/L) | 22±11 | 21±9 | 26±22 | 0.36 | / |
| ALT (IU/L) | 18±8 | 20±12 | 24±15 | 0.06 | / |
| γGT (UI/L) | 22±14 | 24±13 | 29±21 | 0.24 | / |
| *Prevalence* | | | | | |
| Gender (women, n; %) | (43) 86 | (32) 63 | (23) 45 | <0.001 | / |
| Smokers (%) | 32 | 27 | 23 | 0.34 | / |
| MI Physical activity (%) | 49 | 45 | 56 | 0.45 | / |
| Obesity (%) | 34 | 51 | 47 | 0.10 | / |
| Hypertension (%) | 72 | 72 | 69 | 0.70 | / |
| Antihypertensive agents (%) | 74 | 67 | 69 | 0.56 | / |
| Hyperlipidemia (%) | 50 | 57 | 53 | 0.77 | / |
| Lipid-lowering agents (%) | 44 | 39 | 37 | 0.49 | / |
| T2DM (%) | 16 | 18 | 20 | 0.63 | / |
| Oral antidiabetic agents (%) | 11 | 10 | 15 | 0.57 | / |
| BMI, body mass index; HG, hand grip; ASMM, appendicular skeletal muscle mass; TC, total cholesterol; AST, aspartate aminotransferase; ALT, alanine aminotransferase, γGT, gamma glutamyltransferase; MI, Moderate-intensity; T2DM, type 2 diabetes mellitus. | | | | | |

| **Supplemental Table 7.** *Mean ± SD participants’ energy, nutrients and food groups intake across tertiles of Mediterranean pattern* *in the elderly population* | | | | | |
| --- | --- | --- | --- | --- | --- |
| Variables | Tertile 1  (low level)  (n=50) | Tertile 2  (medium level) (n=51) | Tertile 3  (high level)  (n=51) | *p-value* | *P-post hoc analysis* |
| Energy intake (kcal/day) | 1577±366 | 1917±585 | 2224±569 | <0.001 | 1 vs 2 0.004  1 vs 3 <0.001  2 vs 3 0.009 |
| Animal protein (g/day) | 37±16 | 46±19 | 45±22 | 0.032 | 1 vs 3 0.042 |
| Plant protein (g/day) | 20±5 | 27±8 | 37±9 | <0.001 | 1 vs 2 <0.001  1 vs 3 <0.001  2 vs 3 <0.001 |
| Animal lipids (g/day) | 25±14 | 29±15 | 26±15 | 0.44 | / |
| Plant lipids (g/day) | 45±17 | 48±23 | 58±25 | 0.013 | 1 vs 3 0.014 |
| Saturated fatty acids (g/day) | 21±8 | 23±10 | 23±9 | 0.40 | / |
| Monounsaturated fatty acids (g/day) | 39±13 | 42±18 | 46±18 | 0.10 | / |
| Polyunsaturated fatty acids (g/day) | 9±4 | 10±5 | 13±7 | 0.005 | 1 vs 3 0.005 |
| Cholesterol (mg/day) | 192±105 | 222±105 | 228±101 | 0.17 | / |
| Carbohydrates (g/day) | 174±44 | 223±67 | 284±72 | <0.001 | 1 vs 2 <0.001  1 vs 3 <0.001  2 vs 3 <0.001 |
| Soluble fiber (g/day) | 2±1 | 3±1 | 5±2 | <0.001 | 1 vs 2 <0.001  1 vs 3 <0.001  2 vs 3 <0.001 |
| Insoluble fiber (g/day) | 6±2 | 9±3 | 13±4 | <0.001 | 1 vs 2 <0.001  1 vs 3 <0.001  2 vs 3 <0.001 |
| Iron (mg/day) | 8±2 | 10±3 | 13±4 | <0.001 | 1 vs 2 <0.001  1 vs 3 <0.001  2 vs 3 <0.001 |
| Calcium (mg/day) | 703±275 | 945±411 | 924±315 | 0.001 | 1 vs 2 0.001  1 vs 3 0.004  2 vs 3 <0.001 |
| Sodium (mg/day) | 1573±653 | 1823±781 | 1953±983 | 0.06 | / |
| Potassium (mg/day) | 2243±632 | 3029±616 | 3893±1000 | <0.001 | 1 vs 2 <0.001  1 vs 3 <0.001  2 vs 3 <0.001 |
| Zinc (mg/day) | 7±2 | 9±3 | 10±3 | <0.001 | 1 vs 2 <0.001  1 vs 3 <0.001 |
| Magnesium (mg/day) | 178±46 | 244±48 | 315±73 | <0.001 | 1 vs 2 <0.001  1 vs 3 <0.001  2 vs 3 <0.001 |
| Selenium (mg/day) | 32±22 | 32±15 | 39±26 | / | / |
| *Food groups* | | | | | |
| Milk and dairy products (servings/day) | 1.4±0.9 | 1.8±0.9 | 1.9±1.2 | 0.048 | / |
| Meat, fish and eggs (servings/day) | 1.2±0.7 | 1.3±0.7 | 1.5±1.1 | 0.22 | / |
| Legumes (servings/day) | 0.1±0.1 | 0.2±0.2 | 0.3±0.2 | <0.001 | 1 vs 3 <0.001  2 vs 3 0.021 |
| Cereals (servings/day) | 2.4±0.9 | 2.9±1.4 | 3.7±1.3 | <0.001 | 1 vs 3 <0.001  2 vs 3 0.007 |
| Vegetables (servings/day) | 1.3±0.7 | 1.6±0.8 | 2.3±1.0 | <0.001 | 1 vs 3 <0.001  2 vs 3 <0.001 |
| Fruit (servings/day) | 1.1±0.7 | 2.0±1.1 | 2.8±1.3 | <0.001 | 1 vs 2 <0.001  1 vs 3 <0.001  2 vs 3 0.002 |
| Oils/Animal fats/margarines (servings/day) | 1.8±0.8 | 1.9±1.2 | 2.2±1.1 | 0.15 | / |
| Cakes/pies (servings/day) | 1.7±1.6 | 1.8±1.2 | 1.9±1.2 | 0.72 | / |
| Adherence to the MD (score) | 29±2 | 30±3 | 31±3 | <0.001 | 1 vs 3 <0.001 |
| MD, Mediterranean Diet. | | | | | |
